# Back to Front in *C. elegans*

**DOI:** 10.1371/journal.pbio.0040426

**Published:** 2006-11-21

**Authors:** Rachel Jones

An enduring question in biology is how a single cell—the fertilized egg, or zygote—can generate the incredible complexity and variety of tissues of a complete organism. Much of this process depends on the establishment and maintenance of polarity, both in single cells and within a population of cells. Simply put, how does a cell or an embryo know its front from its back?

In a new study, Marcus Bischoff and Ralf Schnabel show that the polarization of the *Caenorhabditis elegans* embryo depends on a “polarization center” that is formed by the descendants of one of the two initial blastomeres—the cells that result from the first division of the zygote. The posterior blastomere gives rise to a group of cells that are responsible for the anterior–posterior organization of most of the embryo.

It has been clear for some time that posterior cells in the early *C. elegans* embryo could influence the orientation of their anterior neighbors and that this influence depends on the signaling molecule Wnt. Bischoff and Schnabel used experiments in which they cultured different combinations of blastomeres to investigate the extent and mechanisms of this effect.

When the anterior blastomere (AB) is isolated from the posterior (P1) cell at the two-cell stage, the dividing cells form a spherical shape. Each cell divides along a cleavage axis; in cultures of isolated anterior blastomeres, the direction of cleavage rotates by about 90 ° after each division, and the divisions fall along an axis that deviates from the anterior–posterior axis by about 63 °. The embryonic fragments formed by this process are spherical. In normal embryos, the descendants of AB cleave along an axis that falls about 45 ° away from the anterior–posterior axis, and the embryos are elongated.

When the authors added a P2 blastomere—one of the descendants of P1—to the isolated anterior cells, it caused a shift in the orientation of division toward the P2 cell, even in those anterior blastomeres that didn’t touch the added cell. It also caused the spherical embryonic fragments to elongate, and the elongation and shift in orientation of division were highly correlated. Further analysis showed that the elongation was a direct result of the reorientation of the cell divisions by P2.

To investigate the role of Wnt signaling in this process, Bischoff and Schnabel carried out similar experiments using blastomeres from embryos with mutations in the gene that encodes the MOM-2/Wnt signaling molecule. When the added P2 cell lacked Wnt, the anterior cells failed to elongate or to orient toward P2. The same effect was seen when the AB-derived blastomeres lacked the Wnt receptor MOM-5/Frizzled, showing that P2 induces reorientation and elongation by producing Wnt, which stimulates Frizzled receptors on AB-derived cells.

**Figure:**
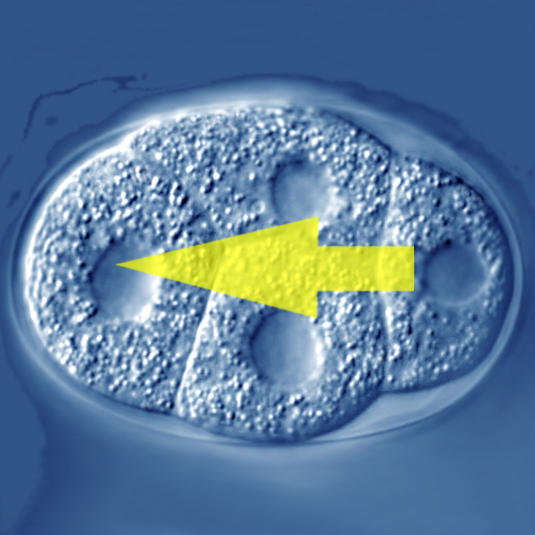
The posterior P2 blastomere polarizes the four-cell embryo, thereby organizing the cleavage directions of cells—which shapes the embryo and directs cell-fate determination.

The properties of this polarizing center were tested using various arrangements of blastomeres. When two AB blastomeres were added to opposite sides of one P2 cell, both oriented toward P2, showing that it signals in all directions. P2 can orient the granddaughter cells of AB as well as the daughter cells, and the daughter cells of P2 have the same effect. Furthermore, the polarizing signal can reach cells that do not touch P2. If the intervening AB descendants lack the ability to produce Wnt, however, the signal does not reach the cells that are not touching P2, showing that the polarizing Wnt signal is transduced from cell to cell by a relay mechanism—each cell, when stimulated by Wnt, in turn produces Wnt to stimulate its neighbors. To confirm this finding, the authors showed that AB cells that had been oriented by a P2 cell could orient other AB descendants even when the P2 blastomere had been removed. This is the first demonstration that cell polarity is passed on from cell to cell by a so-called relay mechanism.

This simple mechanism could have parallels in other organisms, such as *Drosophila* and vertebrates, where it is also necessary to organize polarity in fields of cells. Further work will also be needed to investigate how this mechanism is related to the initial anterior–posterior organization of the zygote by *par* genes, which give rise to the initial distinction between anterior and posterior cells.

